# Mixotrophic chain elongation with syngas and lactate as electron donors

**DOI:** 10.1111/1751-7915.14163

**Published:** 2022-11-15

**Authors:** Flávio C. F. Baleeiro, Jana Raab, Sabine Kleinsteuber, Anke Neumann, Heike Sträuber

**Affiliations:** ^1^ Department of Environmental Microbiology Helmholtz Centre for Environmental Research – UFZ Leipzig Germany; ^2^ Technical Biology, Institute of Process Engineering in Life Science Karlsruhe Institute of Technology – KIT Karlsruhe Germany

## Abstract

Feeding microbial communities with both organic and inorganic substrates can improve sustainability and feasibility of chain elongation processes. Sustainably produced H_2_, CO_2_, and CO can be co‐fed to microorganisms as a source for acetyl‐CoA, while a small amount of an ATP‐generating organic substrate helps overcome the kinetic hindrances associated with autotrophic carboxylate production. Here, we operated two semi‐continuous bioreactor systems with continuous recirculation of H_2_, CO_2_, and CO while co‐feeding an organic model feedstock (lactate and acetate) to understand how a mixotrophic community is shaped during carboxylate production. Contrary to the assumption that H_2_, CO_2_, and CO support chain elongation via ethanol production in open cultures, significant correlations (*p* < 0.01) indicated that relatives of *Clostridium luticellarii* and *Eubacterium aggregans* produced carboxylates (acetate to *n*‐caproate) while consuming H_2_, CO_2_, CO, and lactate themselves. After 100 days, the enriched community was dominated by these two bacteria coexisting in cyclic dynamics shaped by the CO partial pressure. Homoacetogenesis was strongest when the acetate concentration was low (3.2 g L^−1^), while heterotrophs had the following roles: *Pseudoramibacter*, *Oscillibacter*, and *Colidextribacter* contributed to *n*‐caproate production and *Clostridium tyrobutyricum* and *Acidipropionibacterium* spp. grew opportunistically producing *n*‐butyrate and propionate, respectively. The mixotrophic chain elongation community was more efficient in carboxylate production compared with the heterotrophic one and maintained average carbon fixation rates between 0.088 and 1.4 g CO_2_ equivalents L^−1^ days^−1^. The extra H_2_ and CO consumed routed 82% more electrons to carboxylates and 50% more electrons to carboxylates longer than acetate. This study shows for the first time long‐term, stable production of short‐ and medium‐chain carboxylates with a mixotrophic community.

## Graphical abstract

A mixotrophic microbial community able to produce C2–C8 carboxylates from syngas, lactate, and acetate was enriched and cultivated for 292 days. The community was stable in the long term and could produce 50% more C > 2 carboxylates by sourcing extra electrons and carbon from syngas.

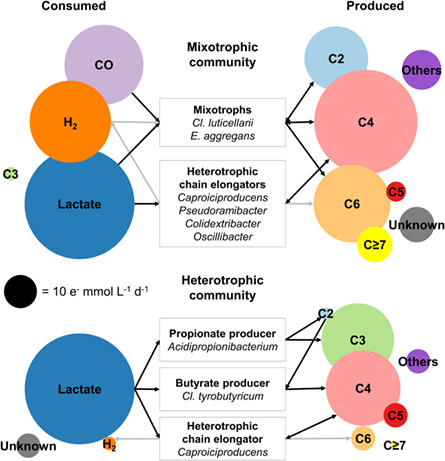

## INTRODUCTION

The production of medium‐chain carboxylates (MCCs) via anaerobic fermentation with microbial chain elongation was initially developed with heterotrophic bacteria. The first chain‐elongating bacteria isolated were cultured with ethanol (*Clostridium kluyveri*) (Schoberth & Gottschalk, 1969) or lactate (*Megasphaera elsdenii*) (Elsden & Lewis, 1953) as electron donors and produced MCCs as well as H_2_ and CO_2_. Bacteria like these inspired seminal work with mixed microbial communities using continuous reactors fed with organic substrates rich in electron donors. Consequently, these studies revealed highly specialized heterotrophic communities (Agler et al., 2014; Grootscholten et al., 2013; Sträuber et al., 2012).

Nowadays, there is renewed interest in the production of MCCs (e.g., *n*‐caproate and *n*‐caprylate) via anaerobic fermentation, as they already have established markets as specialty chemicals (Dessi et al., 2021) currently supplied by unsustainable production routes (McDowall et al., 2022) and are promising precursors of drop‐in fuels (Urban et al., 2017). In terms of sustainability and economic feasibility, there is a strong incentive to develop processes with autotrophic chain elongation communities. Inorganic substrates such as H_2_, CO_2_, and CO (syngas) can be supplied from various green conversion technologies or industrial off‐gases and are used by autotrophic bacteria to form acetyl‐CoA, which is a key intermediate in chain elongation. Some research groups quickly recognized this potential and conducted several studies on the enrichment of autotrophic chain elongation communities (Esquivel‐Elizondo et al., 2017; Ganigue et al., 2016; Zhang et al., 2013). However, microbial communities face several thermodynamic and kinetic bottlenecks when MCCs are produced by autotrophic metabolism (González‐Cabaleiro et al., 2013), and product concentrations and productivities are much lower than those of heterotrophic communities (Baleeiro et al., 2019). Therefore, the solution to make MCC production more feasible and sustainable may lie between pure heterotrophy and pure autotrophy.

Co‐feeding both organic and inorganic substrates to a microbial community with the aim of developing a mixotrophic process is not a straightforward task. In general, the consumption of organic substrates that generate ATP via substrate‐level phosphorylation, such as sugars, is preferred over inorganic substrates via catabolite repression (Gorke & Stulke, 2008). Moreover, long‐term community enrichment often selects for microorganisms that can produce the most cell mass (Liu, Kleinsteuber, et al., 2020), causing desired community functions such as autotrophy or chain elongation to be sidelined. A possible solution to this problem was presented by Park et al. (2019), who co‐fed small amounts of glucose as a “dopant” during autotrophic growth of *Moorella thermoacetica* at constantly high H_2_/CO_2_ availability. Park et al. (2019) observed that yield and productivity of acetate increased with decreasing amounts of glucose. However, a minimum amount of glucose was essential to provide just enough ATP and NADPH to overcome metabolic bottlenecks of autotrophic growth and balance fluxes of reductive acetyl‐CoA metabolism. Recently, several studies adopted the strategy of co‐feeding mixtures of H_2_, CO_2_, and/or CO with organic feedstocks and succeeded in developing mixotrophic chain elongation processes (Baleeiro, Kleinsteuber, & Sträuber, 2021; Esquivel‐Elizondo et al., 2018; González‐Tenorio et al., 2020; Liu, Wang, et al., 2020; Wu et al., 2019). These studies reported improvements in carboxylate production due to co‐feeding as it allowed routing more electrons into longer‐chain carboxylates. However, the community structures and functions that enable this synergy are still poorly understood.

The continuous gas recirculation system presented previously (Baleeiro, Kleinsteuber, & Sträuber, 2022) provides conditions similar to those presented by Park et al. (2019) to favour reductive metabolism as it can be operated with abundant syngas availability and limited organic substrate feeding. In the present study, we hypothesized that the improvement in reductive metabolism could be exploited in chain elongation by using the gas recirculation system to develop a mixotrophic chain elongation process with a microbial community growing on inorganic (H_2_, CO_2_, and CO) and organic (lactate and acetate) substrates. First, the two reactors harbouring communities with differing degrees of specialization were operated in parallel. The best‐performing community was selected to be used in further experiments. Afterwards, one reactor was used as a control while the other was subjected to changes in operating conditions. This way, we aimed to understand the dynamics of the mixotrophic community and to identify its key players during its adaptation and stable phases in long‐term operation.

## EXPERIMENTAL PROCEDURES

### Bioreactor operation

Two identical Minifors reactors (INFORS AG) with a working volume of 1.0 L each and equipped with gas recirculation systems were operated inside a fume cupboard. Each reactor had a peristaltic pump (model 323; Watson Marlow Ltd.) operating continuously to recirculate a syngas mixture (32% H_2_, 32% CO, 16% CO_2_, and 20% N_2_) from its gas reservoir to the broth via microspargers at a rate of 40 ml min^−1^. Reactor 1 and Reactor 2 were operated at 32°C and a pH value of 6.0 ± 0.1 in a semi‐continuous manner with harvesting and feeding realized every 1, 3.5, or 4 days, depending on the experimental phase. Details of the system, the materials, the gas reservoir replenishment, and the measures to inhibit methanogens can be found in the Appendix S1. An illustrative scheme and balance calculations were provided previously by Baleeiro, Kleinsteuber, and Sträuber (2022).

The growth medium was formulated to represent a feedstock limited in electron donors, such as corn silage, which generates an excess of acetate (Lambrecht et al., 2019). It contained 133 mM lactate (12 g L^−1^) as organic electron donor and 200 mM acetate (12 g L^−1^) as organic electron acceptor and has been used in our previous experiments with the gas recirculation reactors (Baleeiro, Ardila, et al., 2021; Baleeiro, Kleinsteuber, & Sträuber, 2022). The complete composition of the growth medium is shown in Table S1. A description of the preparation, handling, and storage of the medium is available in the Appendix S1.

At start‐up, each reactor was inoculated with a different microbial community. Reactor 1 received 50 vol% sludge from a mesophilic biogas reactor and 50 vol% lactate‐free growth medium with 400 mM acetate. Before being used, the sludge was stored overnight for sedimentation to reduce the amount of solids added with the inoculum. Reactor 2 received 100 vol% broth of an enrichment culture able to produce ca. 4 g L^−1^ *n*‐caproate from H_2_/CO_2_, lactate, and acetate under similar conditions (Baleeiro, Ardila, et al., 2021). Additional details on the origin and conditions of the inocula are available in the Appendix S1.

In total, the two reactors were operated for 292 days with the first 61 days being used to compare the two different communities. On day 61, the enriched community of Reactor 2 was discarded and the diverse community from Reactor 1 was distributed to both reactors to continue the experiments comparing three operational parameters in succession (Figure S1). From day 110 to 148, different feeding intervals (1 days vs. 4 days) were compared, from day 181 to 261, the effect of acetate in the feed was investigated (200 mM acetate vs. acetate‐free medium), and from day 272 to 292, different hydraulic retention time (HRT) values were compared (14 days vs. 10 days). Each comparison phase was preceded by a stabilization period for both reactors, which was sufficient to reach a similar state in terms of carboxylate concentration. Average production (+) or consumption (−) rates were calculated for the comparison phases only.

### Chemical analyses

To analyse the carboxylates and alcohols in each reactor, about 1.5 ml of broth was collected three times a week before feeding. Exceptionally, Reactor 1 was sampled on day 42 every 2–3 h over one feeding cycle. High‐performance liquid chromatography (HPLC) with a refractive index detector was used to quantify the concentrations of carboxylates and alcohols, while carboxylates were redundantly quantified via HPLC‐UV at a wavelength of 280 nm. Gas samples were collected with every liquid sampling and always before and after the replenishment of the gas reservoir. Gas chromatography (GC) with a temperature conductivity detector was used to monitor the composition of gases. Details of the sample preparation procedures and configuration of the HPLC and GC systems have been described previously (Baleeiro, Varchmin, et al., 2022). Biomass concentration was monitored via optical density at 600 nm (OD_600_) assuming 0.455 g_dry mass_ L^−1^ per OD_600_ unit (Baleeiro, Kleinsteuber, & Sträuber, 2021). Data tables with the conversion factors used for carbon and electron balances, average rates, CO_2_ balance estimates, as well as the original abiotic data used in this study are available in spreadsheet format in the Data S1.

The formula for the carbon fixation rate and assumptions for estimating the sources of CO_2_ emissions can be found in the Appendix S1.

### Microbial community analysis

About 6.0 ml of broth was collected from each reactor twice per week, always before feeding the reactors, to monitor the microbial community composition. Amplicons of the V3–V4 region of the 16S rRNA gene were sequenced using the Illumina MiSeq platform. The resulting reads were filtered and denoised using the DADA2 workflow (Callahan et al., 2016), and the taxonomy of the resulting amplicon sequence variants (ASVs) was assigned using the SILVA 138 database (Yilmaz et al., 2014). All samples were rarified to an equal depth of 31,892 read counts, which corresponded to the number of reads in the sample with the lowest read number. Details of the wet‐lab protocols such as DNA extraction, primers, and PCR conditions were described by Logroño et al. (2020), while information on the library preparation was given by Baleeiro, Kleinsteuber, and Sträuber (2021). The raw, adapter‐free sequence data for this study has been deposited in the European Nucleotide Archive (ENA) under the study accession number PRJEB52337 (http://www.ebi.ac.uk/ena/data/view/PRJEB52337). Details on the clustering technique used in this study are given in the Appendix S1. Spearman correlations were obtained between the relative ASV abundance and the rates of chemicals calculated between two consecutive sampling points. To decrease the noise, only rates from sampling intervals of at least 1 day were considered. 168 samples containing both adequate rate values and information on relative ASV abundance were used in the correlation analysis.

## RESULTS AND DISCUSSION

### Start‐up with different microbial communities

For the start‐up, the two reactors were operated with an HRT of 14 days and fed with a mineral medium containing 200 mM acetate and 133 mM lactate once a day. Reactor 1 received an inoculum with a diverse community (richness of 392 ASVs), while Reactor 2 received an inoculum with an enriched community (26 ASVs). The microbial composition of the inocula is shown in Figure S2.

**FIGURE 1:**
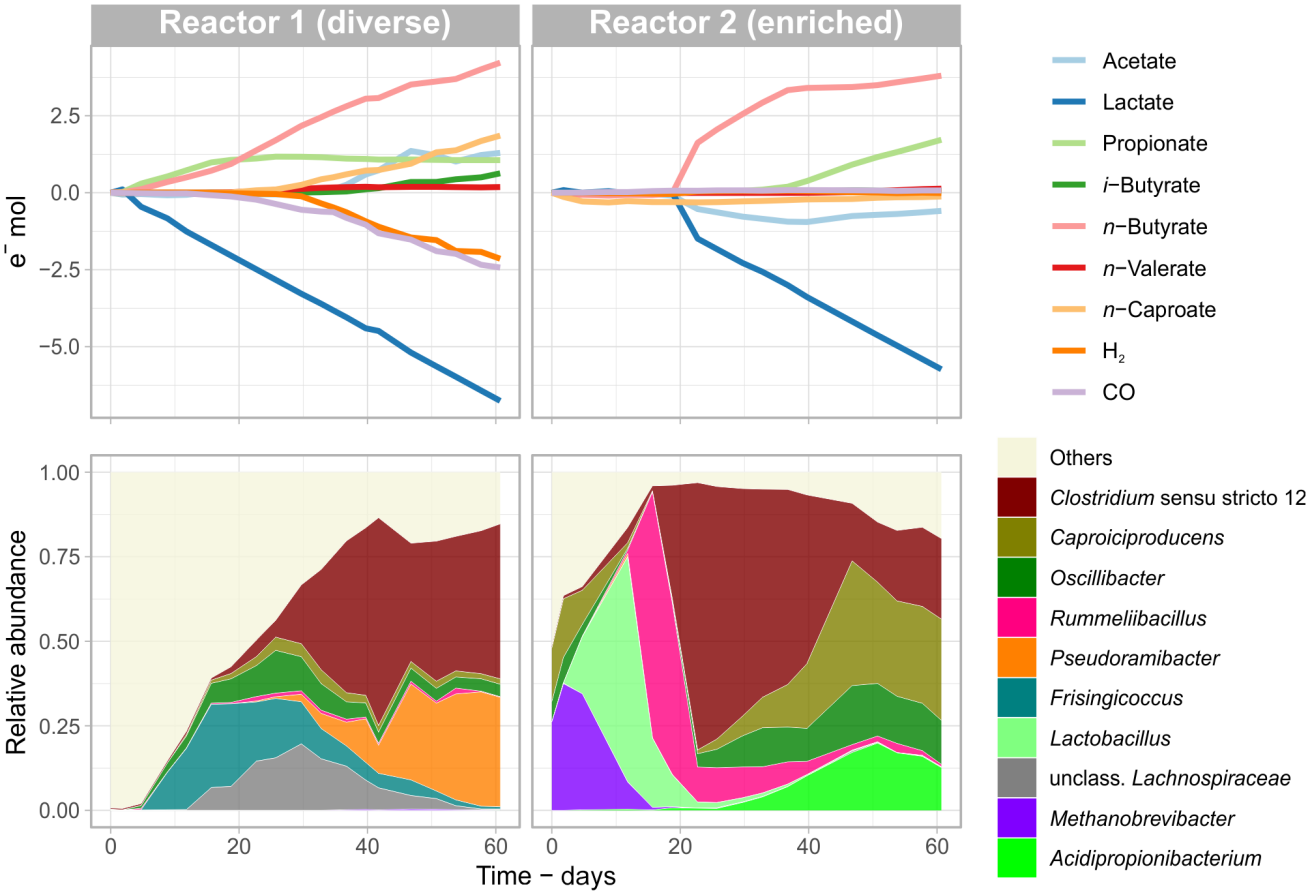
Cumulative electron profiles of chemicals and community composition at the genus level during the first 61 days of fermentation. A diverse community (reactor 1) and an enriched community (reactor 2) were compared. The 10 most abundant genera during the period are shown.

The diverse community in Reactor 1 had a short lag phase of 2 days, after which lactate consumption started concomitantly with the production of propionate and *n*‐butyrate, as shown in Figure 1. Propionate production halted on day 16, enabling more *n*‐butyrate to be formed. Gas consumption started on day 19, initially due to CO consumption, and accelerated after day 30 with simultaneous consumption of H_2_ and CO. Also on day 30, *n*‐caproate production started, coinciding with increasing relative abundances of *Clostridium* sensu stricto 12 and *Pseudoramibacter*. On day 33, in addition to the 12 g L^−1^ acetate already present in the feed, more acetate was produced concurrently with the consumption of H_2_ and CO. Kinetic sampling over a feeding cycle of Reactor 1 revealed that all lactate was consumed within the first 8 h while H_2_ and CO continued to be consumed during the whole 24‐h cycle (Figure S3). Accumulation of acetate stopped suddenly on day 47, after reaching a peak of 19 g L^−1^ (Figure S4).

In Reactor 2, 19 days passed before the enriched community adapted to CO and started producing carboxylates (Figure 1). During the first days, the lack of microbial activity left the system in a relatively oxidized state (oxidation reduction potential, −112 mV), giving the broth the characteristic pink colour of oxidized resazurin. Consequently, the period between days 2 and 19 presented very low biomass concentrations (OD_600_ of 0.07) and high relative abundances of facultative anaerobes *Lactobacillus* and *Rummeliibacillus* (Figure 1). On day 19, consumption of lactate and acetate and concomitant formation of *n*‐butyrate started. On day 37, *n*‐butyrate production slowed down and was replaced by propionate formation, most probably by *Acidipropionibacterium*, resulting in a propionate‐to‐acetate ratio of 2:1.

Despite the high relative abundance of *Caproiciproducens* in the enriched community, *n*‐caproate was not produced during the first 61 days of operation of Reactor 2. Further, no consumption of CO or H_2_ was observed despite the high relative abundance of *Clostridium* sensu stricto 12, a genus that harbours many homoacetogens. These observations are discussed below in the section Clostridial community dynamics.

### Effect of changing operation conditions on process performance

The better‐performing diverse community was transferred to both reactors on day 61. In the following days (61–148 days), the community was given time to stabilize and the feeding interval was increased from 1 to 4 days in Reactor 1 (Figure S1). Increasing the feed interval had minimal impact on carboxylate production and community composition (see detailed description in the Appendix S1 and Figure S5). In fact, the observed community dynamics emerged due to the routine reactor operation rather than the change in feeding regime. Remarkably, cyclic dynamics of *Clostridium* and *Eubacterium* were observed for the first time on day 110 and persisted until the end of reactor operation. These dynamics are examined in more detail below.

**FIGURE 2:**
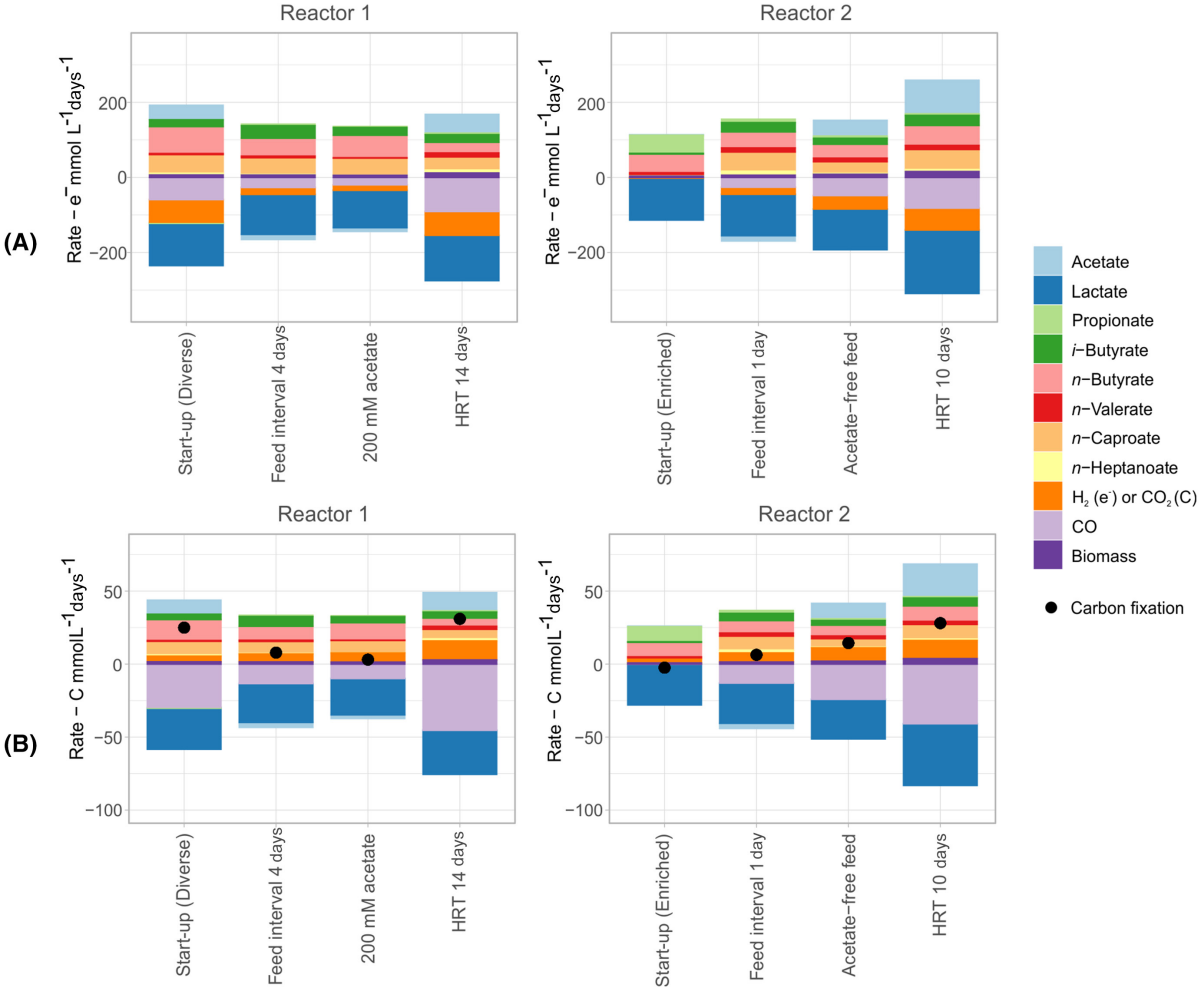
Production (+) and consumption (−) rates in terms of (A) electron equivalents and (B) carbon equivalents for the different conditions tested in the study. Except for the start‐up of reactor 2, the diverse community was used in all tests.

Ceasing the acetate supply of Reactor 2 (181–261 days) and afterwards decreasing its HRT (272–292 days) had substantial effects on the production of carboxylates but not on the community structure at genus level (Figure S6). H_2_ + CO consumption and carbon fixation rates were 2.5‐ and 3.7‐fold higher, respectively, once acetate supply ceased in Reactor 2 in comparison to Reactor 1, which remained supplied with 200 mM acetate (Figure 2A). On the other hand, the production of elongated carboxylates (C ≥ 4) was 25% lower when no acetate was fed. Reducing the HRT from 14 to 10 days resulted in an equivalent increase in the production rates of carboxylates, when comparing Reactors 1 and 2 (Figure 2). Disregarding the operation with the enriched community, the average carbon fixation rate was between 2.0 and 31.0 C mmol L^−1^ days^−1^ (0.088 and 1.4 g CO_2_ equivalents L^−1^ days^−1^, respectively), with the most favourable period observed in Reactor 1 at an HRT of 14 days without acetate supply (Figure 2B). These volumetric carbon fixation rates are comparable with rates observed in state‐of‐the‐art microalgal reactors (Yahya et al., 2020). However, the carbon fixation reported here was due to CO consumption (Figure 2B).

If the assumptions done for assessing the source of the emitted CO_2_ hold true (see Appendix S1), most CO_2_ emissions came from the decarboxylation of lactate that occurs during the conversion of lactate to even‐chain carboxylates and not from the CO oxidation to CO_2_. From the 9.17 mmol CO_2_ L^−1^ days^−1^ expected to have come from lactate decarboxylation by the diverse community during the first comparison phase (day 26–61), 3.77 mmol CO_2_ L^−1^ days^−1^ were abated by CO_2_‐consuming bacteria and 5.41 mmol CO_2_ L^−1^ days^−1^ were emitted. CO_2_ emissions due to CO oxidation were only evident after acetate supply ceased. Without acetate in the feed, the overall carbon fixation rates increased, despite more CO_2_ emissions (Figure 2B). The increase in CO_2_ emission was likely due to CO oxidation to CO_2_ and was estimated to be between 0.83 mmol CO_2_ L^−1^ days^−1^ (Reactor 2, HRT = 10 days, acetate‐free feed) and 6.12 mmol CO_2_ L^−1^ days^−1^ (Reactor 1, HRT = 14 days, acetate‐free feed). Estimates of the source of the emitted CO_2_ for all comparison phases can be found in the Appendix S1. For identifying the pathways responsible for CO_2_ formation with more confidence, isotope techniques could be used.

### Cyclic dynamics of community members

**FIGURE 3:**
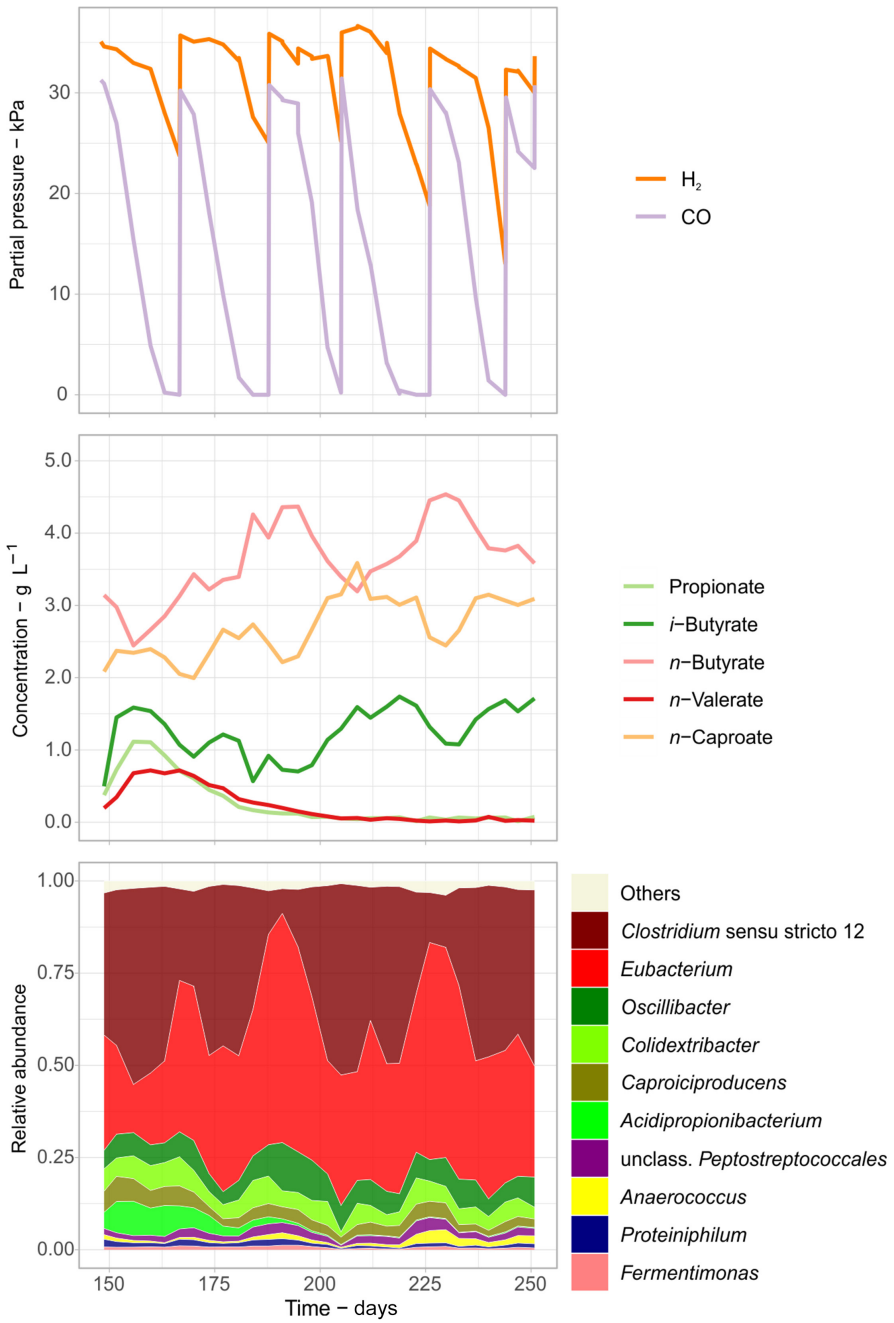
Cyclic dynamics of gas partial pressures, concentrations of carboxylates, and community composition over the longest period with constant operating conditions (Reactor 1, 148–250 days). The 10 most abundant genera are shown.

Although both reactors achieved a stable community composition and chemical output by day 148, relative abundances of *Clostridium* sensu stricto 12 and *Eubacterium* kept cycling oppositely (Figure S6). We observed that at higher CO partial pressures, relative abundances of *Clostridium* sensu stricto 12 increased, whereas *Eubacterium*, *Oscillibacter*, and *Colidextribacter* were more abundant during lower CO partial pressures (Figure 3). Moreover, *n*‐caproate and *i*‐butyrate concentrations varied similarly, but *n*‐butyrate concentration varied in the opposite direction. Peaks of *n*‐caproate did not always coincide with lower or higher partial pressures of CO, hence potential CO inhibition on chain elongation did not seem to be a major concern. Drops in *n*‐butyrate concentration (and simultaneous increases in *n*‐caproate concentration) frequently coincided with increasing relative abundances of *Clostridium* sensu stricto 12, although exceptions were also found.

The dynamics of the community co‐producing *n*‐caproate and *i*‐butyrate (Figure 3) had striking similarities to those found in communities producing *i*‐butyrate from methanol and acetate (de Leeuw et al., 2020; Huang et al., 2020). In these studies, operating conditions such as pH (de Leeuw et al., 2020), use of the methanogenesis inhibitor 2‐bromoethanosulfonate, and cell retention (Huang et al., 2020) defined the competition between *Clostridium* sensu stricto 12 and *Eubacterium* and the product selectivity between *i*‐butyrate and *n*‐butyrate. Differently from here, *n*‐caproate production was not the focus of Huang et al. (2020) nor de Leeuw et al. (2020), and no electron donor with high specificity for *n*‐caproate, such as lactate, was used in their experiments.

Co‐production of *i*‐butyrate and *n*‐caproate was not only observed here but also in reactors fed with H_2_/CO_2_/lactate/acetate (Baleeiro, Ardila, et al., 2021; Baleeiro, Kleinsteuber, & Sträuber, 2022). Therefore, we speculate that another side‐effect of co‐feeding C1 substrates (i.e. CO, H_2_/CO_2_, or methanol) during chain elongation is the expansion of the product spectrum to *i*‐butyrate.

### Functional role of community members

**FIGURE 4:**
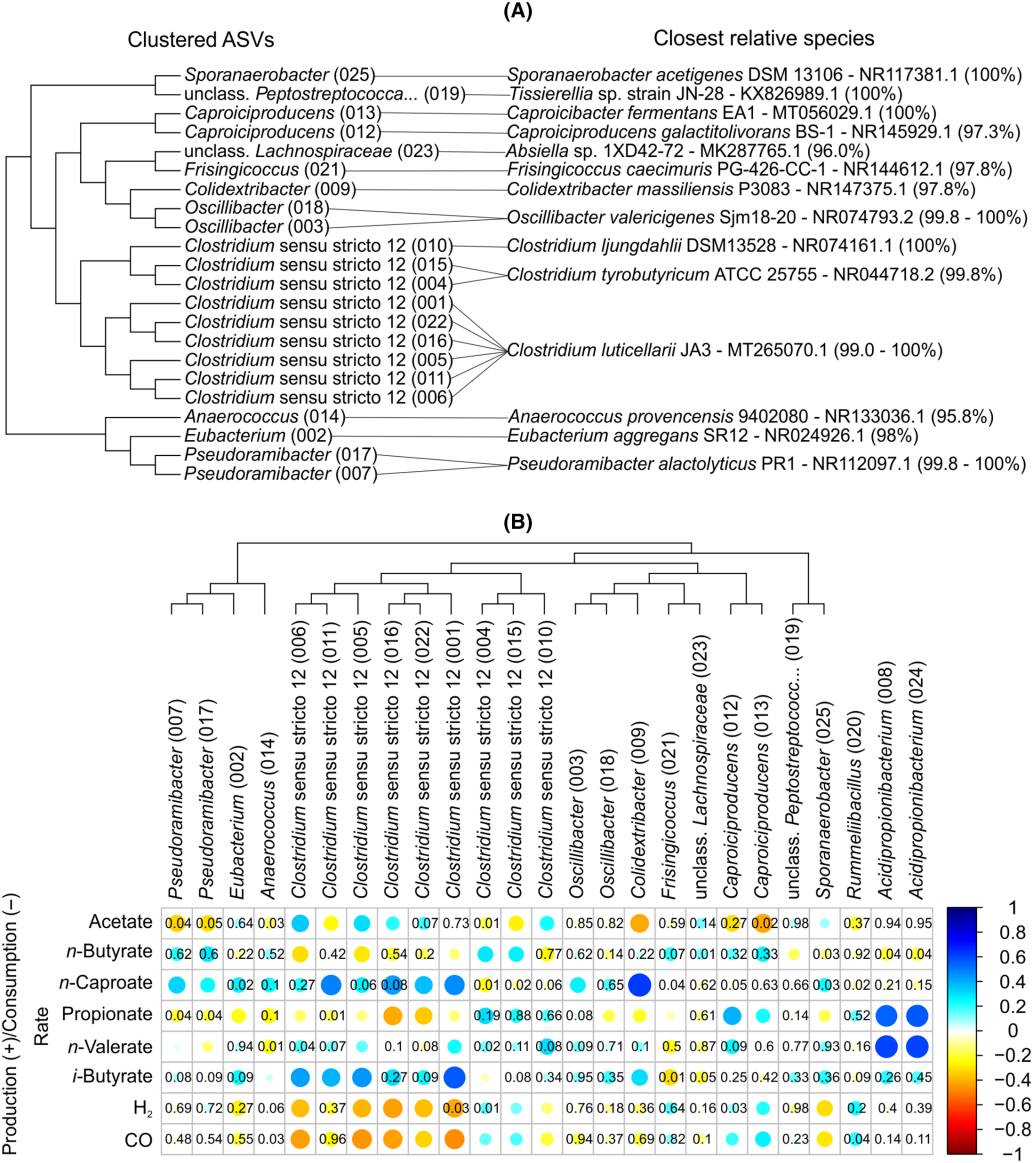
(A) Clustering of the most abundant clostridial amplicon sequence variants (ASVs) with the ASV number in parentheses (left) and their closest cultured relatives with BLAST identities in parenthesis (right). (B) Spearman correlations (*n* = 168) between the 25 most abundant ASVs in the study and the production (+) or consumption (−) rates of chemicals. The *p*‐values are omitted for significant correlations (*p* < 0.01) and shown for correlations with *p* ≥ 0.01.

Analysing the community at the genus level limits the opportunities to deduce potential metabolic functions of community members. In this aspect, the genera *Eubacterium* and *Clostridium* sensu stricto 12 are problematic since they harbour species with very distinct metabolic traits (Kalia et al., 2011; Wade, 2015). To improve the resolution of our community analysis, we performed a cluster analysis of the clostridial ASVs among the 25 most abundant ASVs and did a BLASTN search of these sequences in the NCBI database of 16S ribosomal RNA sequences to find the closest relative species of each. The results are shown in Figure 4A. Additionally, Spearman correlations between relative abundance of the top 25 ASVs and production or consumption rates of the main carboxylates and gases calculated between two consecutive sampling points are shown in Figure 4B.

*Clostridium* sensu stricto 12 contained nine abundant ASVs that had at least 99% similarity to *Cl. tyrobutyricum*, *Cl. ljungdahlii*, or *Cl. luticellarii* (Figure 4A). ASVs 004 and 015 were related to *Cl. tyrobutyricum*, which is a known heterotroph that can consume sugars, lactate, and acetate producing butyrate and H_2_ at pH 6.0 (Fu et al., 2021; Zhu & Yang, 2004). Here, these two ASVs correlated with *n*‐butyrate and H_2_ production (Figure 4B). ASV 010 was a close relative of *Cl. ljungdahlii* (and consequently of its relatives: *Cl. ragsdalei*, *Cl. coskatii*, and *Cl. autoethanogenum*) (Figure 4A), which is mainly known for its autotrophic metabolism able to convert H_2_, CO_2_, and CO into acetate and ethanol (Bengelsdorf et al., 2018). This ASV correlated significantly (*p* < 0.01) to homoacetogenic features, i.e. H_2_ and CO consumption and acetate formation (Figure 4B).

The remaining six *Clostridium* sensu stricto 12 ASVs (ASVs 001, 005, 006, 011, 016, and 022) were related to *Cl. luticellarii* JA3. The type strain *Cl. luticellarii* FW431 has been reported to grow on methanol, H_2_/CO_2_, or lactate while producing a mixture of carboxylates ranging from acetate to *n*‐caproate (Petrognani et al., 2020). Strain JA3 was recently isolated using a *Clostridium* growth medium with glucose and H_2_/CO_2_ (Xu et al., 2020). It has a relatively low 16S rRNA (whole gene) similarity of 97.05% to the type strain and has been designated as a possible new species (Xu et al., 2020). In our dataset, all *Cl. luticellarii* ASVs presented similar correlations (Figure 4B). Most of them showed significant (*p* < 0.01) correlations with H_2_ and CO consumption and with *i*‐butyrate and *n‐*caproate production indicating that these relatives of *Cl. luticellarii* acted as mixotrophic chain elongators in our reactor microbiome, in contrast to the common assumption of interspecies ethanol transfer during gas‐to‐*n*‐caproate formation (Angenent et al., 2016). No information could be found in the literature on a possible carboxydotrophic metabolism (i.e. growth on CO) of *Cl. luticellarii*. Yet, *Cl. luticellarii* has all genes required for the Wood‐Ljungdahl pathway (Poehlein et al., 2018), so carboxydotrophic metabolism is conceivable as suggested by the correlations with CO consumption found here.

*Eubacterium* was represented by a single ASV (ASV 002) with 98% similarity to *E. aggregans* and to *E. barkeri*, which have identical V3–V4 16S rRNA regions. *E. aggregans* and *E. barkeri* are *Eubacterium* sensu stricto that produce acetate and *n*‐butyrate from lactate (Stadtman et al., 1972; Wade, 2015). *E. aggregans* has previously been exploited for its ability to grow on H_2_/CO_2_ producing acetate and *n*‐butyrate, similar to *E. limosum* (Groher & Weuster‐Botz, 2016). The *E. aggregans* strain isolated by Mechichi et al. (1998) does not grow on CO and no autotrophic metabolism was reported for *E. barkeri* (Stadtman et al., 1972). When it comes to growth in the presence of CO, acetogenic *Clostridium* sensu stricto 12 species have higher growth rates than acetogenic *Eubacterium* species (Kang et al., 2020). Therefore, we assume that *Eubacterium* ASV 002 was specialized in H_2_ and lactate consumption in our reactors and could only outcompete *Cl. luticellarii* relatives during periods of low CO partial pressure (Figure 3). Even though increasing relative abundances of ASV 002 coincided with decreasing H_2_ partial pressure (Figure 3), this ASV did not correlate significantly with gas consumption. However, it did correlate with *n*‐caproate formation (*p* = 0.02) (Figure 4B). Hence, the lack of significant correlation of ASV 002 to gas consumption could be due to faster gas consumption rates of *Cl. luticellarii* relatives overshadowing activities of *Eubacterium*, similarly to what we observed previously between *Methanobacterium* and *Clostridium* (Baleeiro, Kleinsteuber, & Sträuber, 2021).

Amplicon sequence variants 007, 012, 013, and 017 were related to known heterotrophic chain elongators (Figure 4A): *Caproicibacter fermentans* (similar to ASV 013), *Caproiciproducens galactitolivorans* (ASV 012), and *Pseudoramibacter alactolyticus* (ASVs 007 and 017) produce *n*‐caproate from sugars (Esquivel‐Elizondo et al., 2020; Kim et al., 2015; Willems & Collins, 2015). Since lactate was the only organic electron donor in our study, these four ASVs might belong to yet uncultured species. In recent studies, *Pseudoramibacter* was among the main suspects of lactate‐based chain elongation (Crognale et al., 2021; Fortney et al., 2021) and a close relative of both *Ca. fermentans* and *Ca. galactitolivorans* able to produce *n*‐caproate from lactate, *Caproicibacterium lactatifermentans*, has been isolated (Wang et al., 2021) and characterized (Wang et al., 2022). Besides, *Candidatus* Pseudoramibacter fermentans is an uncultured species identified by multi‐omics analysis suspected to produce *n*‐caproate from lactate (Scarborough et al., 2020). Our correlation analysis showed no connection between the *Caproiciproducens* relatives and *n*‐caproate formation, while both *Pseudoramibacter* relatives showed significant correlations (Figure 4B). We have previously observed that *n*‐caproate formation by *Caproiciproducens* spp. was inhibited by CO (Baleeiro, Varchmin, et al., 2022), but no studies with *Pseudoramibacter* and CO were found.

*Oscillibacter* (ASVs 003 and 018), *Colidextribacter* (ASV 009), and *Sporanaerobacter* (ASV 025) are genera often found to be abundant in communities producing MCCs (Joshi et al., 2021; Liu et al., 2017; Zagrodnik et al., 2020). Only few isolates of these genera have been characterized. *Oscillibacter valericigenes*, *Colidextribacter massiliensis*, and *Sporanaerobacter acetigenes* are heterotrophs that produce short‐chain carboxylates via sugar fermentation (Hernandez‐Eugenio, 2002; Katano et al., 2012; Ricaboni et al., 2017), with *O. valericigenes* being described additionally as an *n*‐valerate producer. *Oscillibacter* isolates growing autotrophically on H_2_/CO_2_ or CO and producing *i*‐valerate have been reported (Park, Yasin, Kim, Park, et al., 2013; Park, Yasin, Kim, Roh, et al., 2013). Here, *Oscillibacter* and *Colidextribacter* correlated to *n*‐caproate formation and not to gas consumption (Figure 4B), suggesting a heterotrophic chain elongation metabolism. On the other hand, the *Sporanaerobacter* ASV correlated significantly (*p* < 0.01) with homoacetogenic activity.

Other clostridial ASVs had either low similarity to their closest cultured relatives (i.e. ASVs 014 and 023, related to *Anaerococcus provenciensis* 9,402,080 and *Absiella* sp. 1XD42‐72, respectively) or limited literature information on their metabolism (concerning ASVs 019 and 021, related to *Tissierellia* sp. JN‐28 and *Frisingicoccus caecimuris* PG‐426‐CC‐1, respectively). Their roles in the community remained elusive as they presented few significant correlations (Figure 4B).

Expectedly, *Acidipropionibacterium* spp. correlated with propionate formation. What was less expected, however, was the correlation of propionate formation with *Caproiciproducens* spp. (Figure 4B). The *Caproiciproducens* spp. isolated so far were not found to produce propionate (Flaiz et al., 2020). *Caproiciproducens* spp. and *Acidipropionibacterium* spp. often co‐occur (Baleeiro, Ardila, et al., 2021; Kim et al., 2022) since they compete for similar ecological niches in lactate consumption at pH > 5.0 (Kim et al., 2022). Here, the abundances of both genera peaked at about the same time in different experiments (see Figure S5 and Reactor 2 in Figure 1). Therefore, the correlation between *Caproiciproducens* and propionate formation was probably indirect due to the frequent co‐occurrence of *Caproiciproducens* and *Acidipropionibacterium*. Another likely indirect correlation seen in Figure 4B is between *Acidipropionibacterium* spp. and *n*‐valerate production. *n*‐Valerate is a common chain elongation product from propionate and its production is, hence, enhanced by increased propionate production.

### Clostridial community dynamics

**FIGURE 5:**
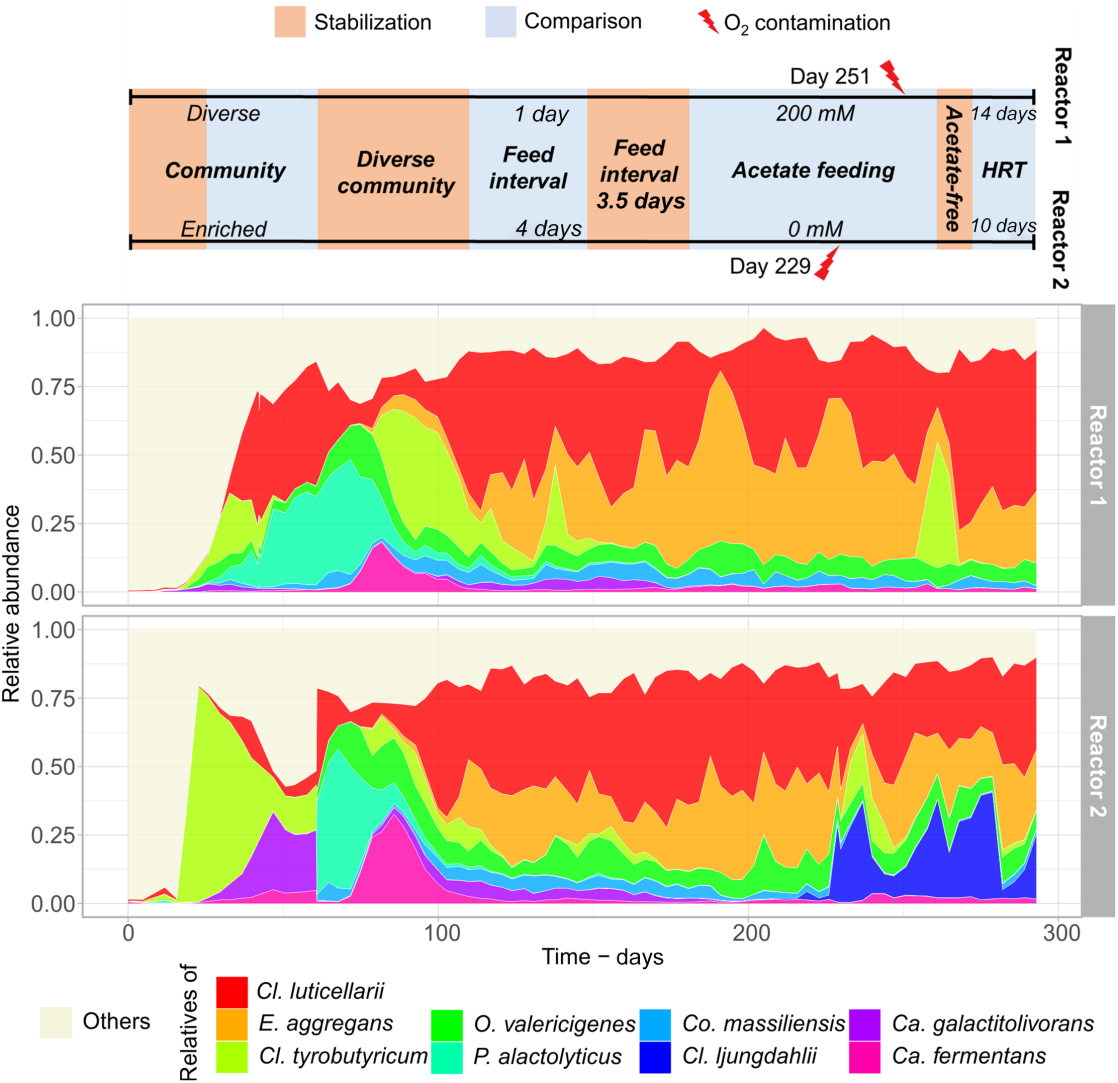
Dynamics of the 10 most abundant clostridial species over the whole reactor experiment. “Others” groups all amplicon sequence variants not assigned to any of these species. The timeline represents the experimental phases in both reactors.

Figure 5 illustrates the community composition over the whole experimental time by grouping the most abundant clostridial ASVs according to their closest relative species. The composition of *Clostridium* sensu stricto 12 below the genus level was fundamentally different in the two reactors during the start‐up period (until day 61). *Cl. luticellarii* relatives were abundant in the diverse community of Reactor 1, whereas relatives of *Cl. tyrobutyricum* predominated in the enriched community of Reactor 2. This difference helps explain the absence of autotrophic and chain elongation activities in Reactor 2 during this period (Figure 1). After the diverse community was distributed to both reactors on day 61, *Cl. tyrobutyricum*, *P. alactolyticus*, and *Ca. fermentans* were outcompeted by *Cl. luticellarii*.

Different dynamics were observed after we changed certain operating conditions. After day 181, acetate supply in Reactor 2 was stopped, causing acetate to be washed out of the broth from a concentration of 10.5 to 3.2 g L^−1^ (Figure S6). Initially, the lower acetate concentration did not have a strong effect on the community composition in Reactor 2, but an unintentional air contamination on day 229 triggered an abrupt increase in the abundances of *Cl. tyrobutyricum* and *Cl. ljungdahlii* at the cost of *Cl. luticellarii* and *E. aggregans* (Figure 5). *Clostridium* species not related to *n*‐caproate production, such as *Cl. ljungdahlii* and *Cl. tyrobutyricum*, were shown to be resistant to low levels of oxygen contamination (Baleeiro, Ardila, et al., 2021). Moreover, *Cl. ljungdahlii* is known for some degree of oxygen tolerance (Whitham et al., 2015). The presence of *Cl. tyrobutyricum* was transient but *Cl. ljungdahlii* remained in Reactor 2 after day 229 until the end of the experiment, coinciding with acetate accumulation again up to 9.4 g L^−1^ on day 272 (Figure S6). The lower acetate concentration in Reactor 2 may have given *Cl. ljungdahlii* the opportunity to establish in the community by occupying the niche of acetate production from H_2_, CO_2_, and CO. Coincidently, Reactor 1 suffered from a similar oxygen shock a few days later (day 251) while having a relatively high acetate concentration of 11.5 g L^−1^ (Figure S6). In this case, the transient abundance of *Cl. tyrobutyricum* ASVs occurred without the increase of *Cl. ljungdahlii* abundance (Figure 5), reinforcing our assumption that the low acetate concentration in Reactor 2 was the cause of the new community structure.

### Mixotrophic efficiency

**FIGURE 6:**
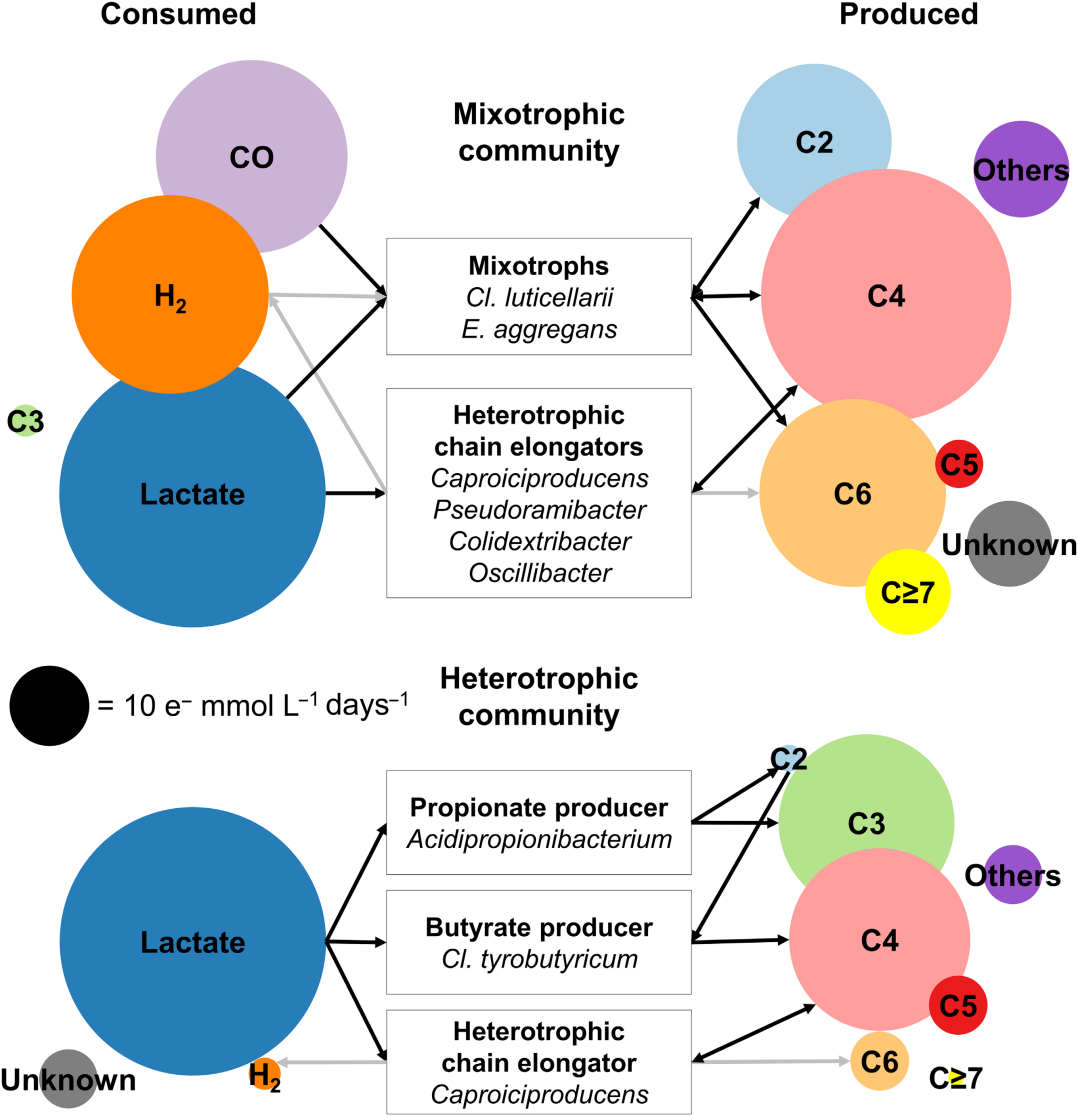
Electron balances in reactor 1 with the mixotrophic community and reactor 2 with the heterotrophic community in the period 26–61 days. Levels of production and consumption rates are proportional to the circular areas. Community members with their putative metabolic function are indicated. Black arrows indicate main fermentation routes while grey arrows indicate pathways that were inhibited by CO. C2: Acetate and ethanol; C3: Propionate and *n*‐propanol; C4: *n*‐butyrate, *i*‐butyrate, and *n*‐butanol; C5: *n*‐valerate, *i*‐valerate, and *n*‐pentanol; C6: *n*‐caproate, *i*‐caproate, and *n*‐hexanol; C ≥ 7: *n*‐heptanoate and *n*‐caprylate; Others: Biomass, formate, CH_4_, and electron losses due to O_2_ contamination; “Unknown” is the difference between the electrons in the consumed and produced pools.

The time window between days 26 and 61 (Figure 1) gave an opportunity to compare a mixotrophic and a heterotrophic community since the enriched community in Reactor 2 consumed virtually no gas. Figure 6 illustrates the electron balances for this period together with putative functions of the most abundant bacterial taxa based on our analyses.

Both communities consumed the same amount of lactate (112 e^−^ mmol L^−1^ days^−1^), however, the mixotrophic community consumed twice as much electron donors since it additionally consumed 119 e^−^ mmol L^−1^ days^−1^, which came in approximately equal shares from H_2_ and CO (Figure 6). The doubling of electron donor consumption by the mixotrophic community reflected an 82% higher production of C2–C8 carboxylates (except lactate) and alcohols, compared with the purely heterotrophic community. The increased consumption did not only enhance the production of acetate (C2), which is a trivial product of syngas fermentation. In fact, the increase in the production of compounds with chains longer than C2 (i.e. C3–C8) due to H_2_ and CO consumption was 50%.

The mixotrophic community produced 11 times more MCCs and alcohols (i.e. C6–C8 compounds) than the heterotrophic community (66.6 and 5.7 e^−^ mmol L^−1^ days^−1^, respectively). Still, this specific comparison should not be extrapolated to all chain elongation communities. The heterotrophic community in Reactor 2 grew in the presence of CO, which is an inhibitor of some chain‐elongating bacteria such as *Clostridium kluyveri* (Diender et al., 2016) and *Caproiciproducens* (Baleeiro, Varchmin, et al., 2022).

Another atypical aspect of the heterotrophic community was that its production of *n*‐butyrate and *n*‐caproate was not accompanied by H_2_ evolution. This was likely due to the presence of CO, which inhibits hydrogenases used by acetogens for H_2_ formation (Menon & Ragsdale, 1996). The heterotrophic community even showed a minor H_2_ consumption (2 e^−^ mmol L^−1^ days^−1^), which might have been caused by scarcely abundant *Cl. luticellarii* relatives (Figure 6). When not inhibited by CO, heterotrophic lactate‐based chain elongation communities route a considerable share of electrons to H_2_ (Brodowski et al., 2021) yielding less carboxylates. In principle, H_2_ can be an interesting by‐product of anaerobic fermentation. In practice, however, H_2_ is readily consumed by methanogens in open cultures (Cabrol et al., 2017) and its separation is challenging at typical concentrations in the headspace of a fermenter (Levin & Chahine, 2010).

We did not observe strong solventogenic activity and the concentration of alcohols remained below 1 g L^−1^ throughout the study. Nevertheless, some *n*‐butanol and *n*‐hexanol was produced by the mixotrophic community (9.4 and 5.2 e^−^ mmol L^−1^ days^−1^, respectively) accounting for ca. 10% of the electron pools of C4 and C6 compounds, respectively. Conversely, alcohol production by the heterotrophic community was negligible. Yet, when alcohol production is the main goal, other bioreactor operation strategies should be taken into account (He et al., 2021) or a chemical conversion route from carboxylates should be considered (Holtzapple et al., 2022).

The mixotrophic community was enriched at relatively high HRT levels (10–14 days), with a feed rich in acetate (200 mM) and poor in lactate (133 mM), and produced a wide spectrum of C ≥ 4 carboxylates and alcohols. Consequently, the production rates of *n*‐caproate reported by us are low in comparison to other studies realizing lactate‐based chain elongation with purely heterotrophic communities. For instance, the maximum *n*‐caproate production rate of 402 e^−^ mmol L^−1^ days^−1^ obtained by Brodowski et al. (2021) using an HRT of 5 days and 300 mM lactate dwarfs the highest *n*‐caproate rates obtained here (up to 54 e^−^ mmol L^−1^ dasy^−1^, Figure 2A). The aspect in which the mixotrophic community shows promise is its efficiency in producing elongated compounds given a fixed amount of organic electron donor. In this sense, the yield of C ≥ 4 compounds obtained with the heterotrophic community enriched by Brodowski et al. (2021) (0.697 e^−^ mmol per e^−^ mmol of lactate) was the half of the yield obtained with the mixotrophic community in this study (1.40 e^−^ mmol per e^−^ mmol of lactate).

## CONCLUSION

The use of microbial communities capable of simultaneously consuming organic substrates, H_2_, CO_2_, and CO is a promising way to make anaerobic fermentation more feasible. By producing more MCCs from a fixed amount of organic substrate, mixotrophic chain elongation alleviates one of the main limitations of fermentation technology, namely the dependence on the cost, quality, and availability of the organic feedstock. More specifically, mixotrophic chain elongation shows promise for processes in which (i) there is commercial interest in not only *n*‐caproate production but also in C ≥ 4 carboxylates and alcohols, (ii) net carbon fixation is desired, and (iii) the supply of the organic feedstock is a limiting factor, given that syngas components can be supplied inexpensively. Relatives of *Cl. luticellarii* and *E. aggregans* composed the core of the stable mixotrophic community and competed for lactate while producing *n*‐caproate. The relative abundances of these bacteria were influenced by the CO partial pressure: *Cl. luticellarii* consumed CO, whereas *E. aggregans* did not. Bacteria with pure heterotrophic metabolism (*Pseudoramibacter*, *Caproiciproducens*, *Colidextribacter*, *Oscillibacter*, *Cl. tyrobutyricum*, and *Acidipropionibacterium*) had either a transient dominance or low abundances in the reactor. High acetate concentrations (ca. 11 g L^−1^) were important to maintain the dominance of mixotrophs but also slowed down carbon fixation. When acetate supply stopped, the production of elongated carboxylates (C ≥ 4) deteriorated and a relative of *Cl. ljungdahlii* seized the opportunity left by lower acetate concentrations and grew autotrophically on syngas, thereby replenishing acetate. To better assess the potential of mixotrophic chain elongation for industrial applications, we recommend applying the concept with real organic feedstocks. Future experiments could unravel the intricate microbial interactions and explain other phenomena we observed, such as the transient dominance of *Pseudoramibacter* or the roles of less abundant community members, by enriching new mixotrophic communities under different conditions. These studies could profit from high‐resolution community analyses such as metagenomics or proteomics.

## AUTHOR CONTRIBUTIONS

**Flávio C. F. Baleeiro:** Conceptualization (equal); data curation (equal); formal analysis (equal); investigation (equal); methodology (equal); visualization (equal); writing – original draft (lead); writing – review and editing (equal). **Jana Raab:** Formal analysis (equal); investigation (equal); writing – review and editing (equal). **Sabine Kleinsteuber:** Conceptualization (equal); formal analysis (supporting); funding acquisition (equal); project administration (equal); resources (equal); supervision (equal); writing – review and editing (equal). **Anke Neumann:** Conceptualization (equal); formal analysis (supporting); funding acquisition (equal); project administration (equal); resources (equal); supervision (equal); writing – review and editing (equal). **Heike Sträuber:** Conceptualization (equal); formal analysis (supporting); funding acquisition (equal); project administration (equal); resources (equal); supervision (equal); writing – review and editing (equal).

## CONFLICT OF INTEREST

The authors declare that the research was conducted in the absence of any commercial or financial relationships that could be construed as apotential conflict of interest.

## FUNDING INFORMATION

This study was funded by the Helmholtz Association, Research Program Renewable Energies. Financial support was also received from the CAPES – Brazilian Coordination for the Improvement of Higher Education Personnel (No. 88887.163504/2018‐00) and from the BMBF – German Federal Ministry of Education and Research (No. 01DQ17016).

## Supporting information


Data S1

Appendix S1
