# Supplementary material for: Mixotrophic chain elongation with syngas and lactate as electron donors
Source: Microb Biotechnol. 2022 Nov 15;16(2):322–36. doi: 10.1111/1751-7915.14163 (PMC9871530; doi:10.1111/1751-7915.14163)
Supplement: Supplementary file 2 — Appendix S1 [file MBT2-16-322-s002.pdf]

Supporting Information

Mixotrophic chain elongation with syngas and lactate as electron donors

Flávio C. F. Baleeiro, Jana Raab, Sabine Kleinsteuber, Anke Neumann, Heike Sträuber

REACTOR OPERATION. 2

CARBON FIXATION AND ASSUMPTIONS FOR ESTIMATING THE SOURCE OF CARBON DIOXIDE EMISSIONS. 2

Table S1. 4

GROWTH MEDIUM PREPARATION 4

INOCULA. 5

ASV CLUSTERING 5

Fig. S1. 6

Fig. S2. 6

Fig. S3. 7

Fig. S4. 7

Fig. S5. 8

Fig. S6. 9

EFFECTS OF CHANGING OPERATING CONDITIONS. 9

REFERENCES 11

REACTOR OPERATION. Neoprene tubes were used for the peristaltic pump heads that worked with liquids, except for the lines that were in contact with the base solution, for which silicon tubes were used. PVC tubing (Tygon® LMT-55, Saint-Gobain Performance Plastics Nagano, Japan) was used for all gas and the remaining liquid lines.

The flexible gas reservoir had a maximum volume of 22 L and the rigid headspace volume of the system was 2.3 L. The gaseous substrate was replenished when one of the following conditions was met: all CO was consumed, the gas reservoir volume was less than 10 L, or the gas bag volume was bigger than 20 L due to net gas production. During replenishment, the empty gas reservoir was filled with 17 L syngas mixture (32% H_2, 32% CO, 16% CO_2, 20% N_2) with 480 mL He injected as a tracer gas and, when applicable, ethylene. Ethylene was used as a methanogenesis inhibitor auxiliary to CO. Since we have previously observed acclimatization of methanogens to ethylene after long exposure (Baleeiro et al. 2021), 2 kPa ethylene (ca. 420 mL) was only used when deemed necessary (between days 47 and 92). This way, methanogenic activity was kept low, with a maximum of 4.4% CH_4 accumulating in the gas phase and an average CH_4 production rate of 11 mL L^{-1} d^{-1}, corresponding to less than 1% of the total organic carbon fed. Air contamination of the systems was monitored by measuring the N_2 content of the gas phase. The details and calculations for monitoring air contamination have been described previously (Baleeiro et al. 2022). Except for two unintended air contamination events during overnight operation on days 229 (Reactor 2) and 251 (Reactor 1), the reactors were kept largely anoxic with an average ORP of −386 ± 73 mV and an average O_2 intrusion rate of 13 mL L^{-1} d^{-1}.

CARBON FIXATION AND ASSUMPTIONS FOR ESTIMATING THE SOURCE OF CARBON DIOXIDE EMISSIONS. The rate of carbon fixation r_{fixed} (in C mmol L^{-1} d^{-1}) was calculated as described in Equation (1).

$$r_{fixed} = -(r_{CH4} + r_{CO2} + r_{CO}) \quad (1)$$

where r_{CH4}, r_{CO2}, and r_{CO} are the production (+) or consumption (−) rates of CH_4, CO_2, and CO, respectively, in C mmol L^{-1} d^{-1}.

In order to estimate whether the CO_2 emissions were due to lactate decarboxylation or CO oxidation to CO_2, CO_2 production rates were first corrected by considering the CO_2 that was consumed for CH_4 and the CO_2 that got washed-out in the medium.

First, we assumed that every for every CH_4 molecule produced, one CO_2 molecule was consumed. The dissolved CO_2 concentration in the medium was estimated to be 11.8 mM CO_2, leading to CO_2 wash-out rates of 0.843 mmol CO_2 L^{-1} d^{-1} (HRT 14d) and 1.18 mmol CO_2 L^{-1} d^{-1} (HRT 10 d). This concentration of

CO_2 in the medium was obtained using Henry's law and the carbonate system equilibria. Assuming 32°C, pH 6.0, and 28.7 kPa CO_2 (average CO_2 partial pressure). Further assumptions for estimating the source of CO_2 emissions were:

1) The amount of decarboxylated lactate (i.e. lactate that emits one CO_2 molecule) can be estimated by subtracting the production rates of all odd-chain compounds from the consumption rates of lactate.

2) Syntrophic carboxylate oxidation was inexistent or negligible.

3) Lactate decarboxylation is a more favorable route for CO_2 emission than CO oxidation.

The corrected CO_2 production rates were subtracted from the amount of decarboxylated lactate to obtain an estimate of the source of CO_2 emissions. If the resulting value was positive, it was interpreted as CO_2 emissions from lactate decarboxylation that were abated by CO_2 fixation. If the resulting value was negative, it was interpreted as evidence for CO_2 being formed via CO oxidation.

Table S1. Growth medium composition. Adapted from Baleeiro et al. (2022).

Concentration of major components - g L^{-1}	
NH_4Cl	1.61
KH_2PO_4	13.6
DL-Lactic acid	12.0
Acetic acid	12.0 (with acetate) or 0.0 (acetate-free)
NaOH (approx.)	11.4 (with acetate) or 7.0 (acetate-free)
**Concentration of minor components - mg L^{-1}**	
$MgCl_2 \times 6\ H_2O$	54
$CaCl_2 \times 2\ H_2O$	65
Resazurin	0.5
Cysteine hydrochloride	30
**Metals, selenium, and boron - µg L^{-1}**	
$FeCl_2 \times 4\ H_2O$	1500
$CuCl_2 \times 2\ H_2O$	2.0
$CoCl_2 \times 6\ H_2O$	190
$MnCl_2$	100
$Na_2MoO_4 \times 2\ H_2O$	36
$NiCl_2 \times 6\ H_2O$	24
$Na_2WO_4 \times 2\ H_2O$	20
$Na_2SeO_3 \times 5\ H_2O$	3.0
$ZnCl_2$	70
H_3BO_3	6.0
**Vitamins - µg L^{-1}**	
Biotin	20
Folic acid	20
Pyridoxine	100
Thiamine	50
Riboflavin	50
Niacin	50
Calcium pantothenate	50
Cobalamin	20
p-Aminobenzoic acid	80
Lipoic acid	50

GROWTH MEDIUM PREPARATION. First, the medium components, except vitamins, cysteine, and NaOH, were dissolved in deionized water under aerobic conditions. The resulting solution was then made anoxic by stirring for at least three hours in an anaerobic chamber. Then, the anoxic solution was transferred into a 2-L borosilicate glass bottle with a two-port cap. The ports of the bottle were kept sealed by crimped silicon tubes during transportation and storage in the anaerobic chamber. Afterwards, the glass

bottle with the solution was sterilized by autoclaving (121ºC for 20 minutes) and stored inside the anaerobic chamber. Vitamins and cysteine hydrochloride were added to the medium in the anaerobic chamber just before connecting the bottle to the reactor system's feed pump. These solutions stemmed from concentrated solutions of vitamins (previously sterilized with a 0.2 µm syringe filter) and cysteine hydrochloride (previously sterilized by autoclaving). Originally, the medium had a pH value of 2.6 (with acetate) or 3.9 (acetate-free). The pH was automatically adjusted to 6.0 by adding 4 M NaOH after being pumped into the reactor. During operation, the salinity of the broth depended on the amount of NaOH added to keep a stable pH value. Therefore, the salinity depended on the total amount of carboxylates in the broth and was estimated to be between 13 and 28 g NaCl eq. L^{-1}.

INOCULA. The reactor from which the diverse community stemmed was a pilot-scale anaerobic digester of the Deutsches Biomasseforschungszentrum (DBFZ, Germany) fed with corn silage and cow manure and operated at 37ºC. The inoculum of the enriched community consisted of broth harvested from the reactor designated as "STR-control" (Baleeiro et al. 2021) until operation day 87. STR-control was operated anoxically at 32ºC and pH 6.0 with an HRT of 14 d and fed with a growth medium similar to the one used here (200 mM acetate, 133 mM lactate). The inocula were stored in closed screw cap plastic bottles at 4 ºC in the dark for about 3 months before being used. On the sampling day of the inoculum, the sludge of the pilot-scale digester had a low carboxylate concentration (343 mg L^{-1} acetate, 58 mg L^{-1} propionate, and 69 mg L^{-1} *n*-butyrate), whereas the broth of the enrichment culture was rich in various carboxylates (9.13 g L^{-1} acetate, 2.53 g L^{-1} *n*-butyrate, 4.62 g L^{-1} *n*-caproate, and 1.21 g L^{-1} *n*-caprylate).

ASV CLUSTERING. Among the 25 most abundant ASVs found in this dataset, the ones assigned to Clostridia (i.e. 22 ASVs) were aligned *de novo* using the library Decipher for R (Wright 2015). The aligned sequences were clustered using the Neighbor Joining model with help of the libraries Phangorn (Schliep et al. 2017) and Ape (Paradis et al. 2004) for R. To identify poor alignment due to short sequences (ASVs were ~400 base pairs long), the sequences were additionally aligned with a reference *E. coli* sequence using the web-based tool SILVA Alignment, Classification and Tree Service (Quast et al. 2013). All alignments had a score of 98 or more.

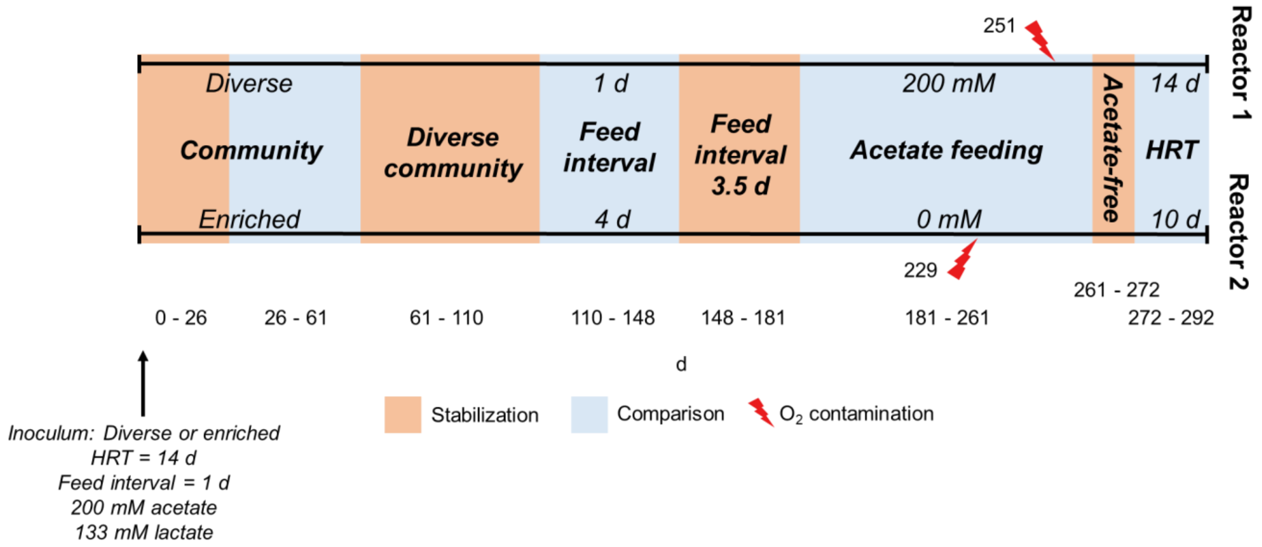

Fig. S1. Summary of the operating conditions in Reactors 1 and 2.

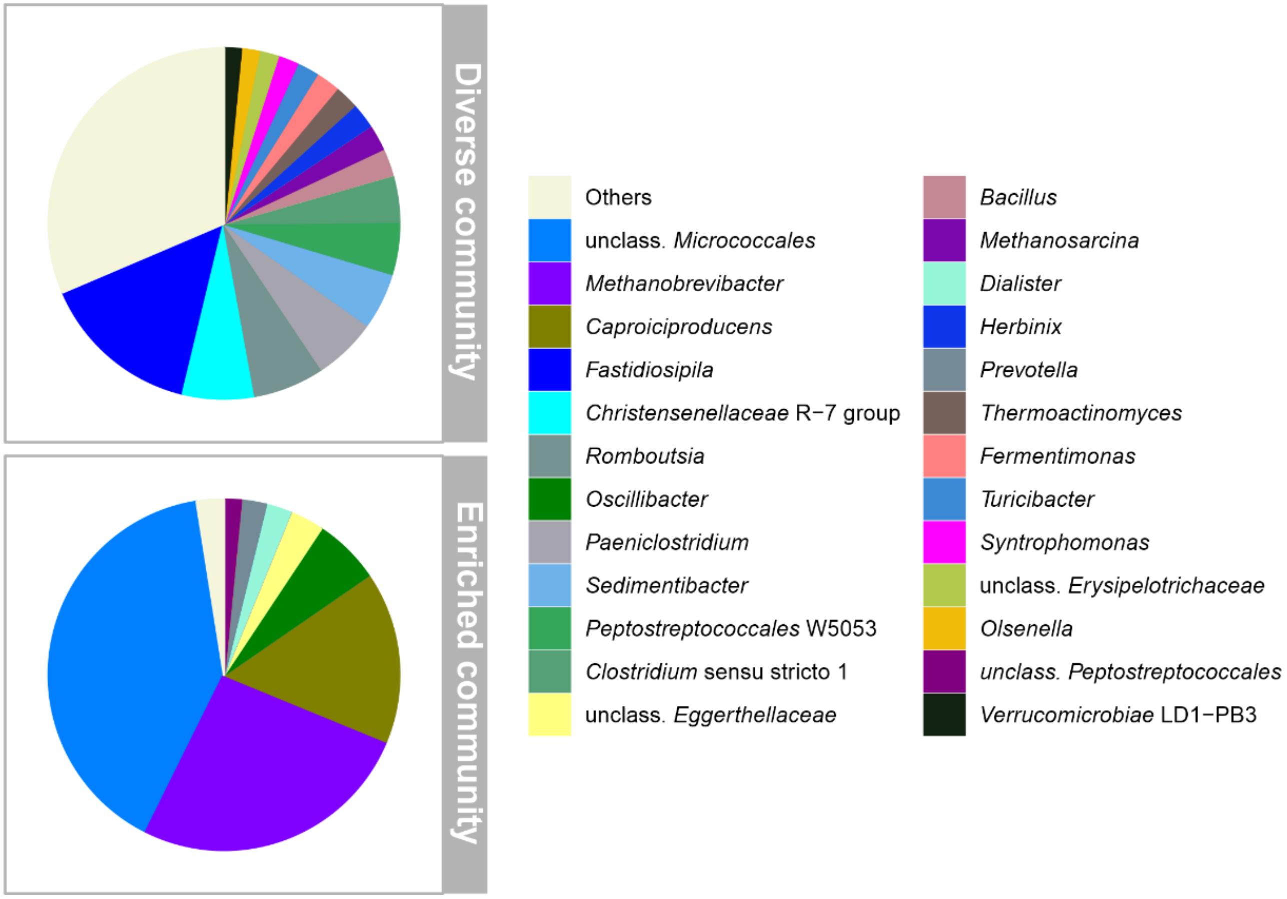

Fig. S2. Microbial community composition of the two inocula used in the study. The 25 most abundant genera in the inocula are shown with the remaining grouped in "Others".

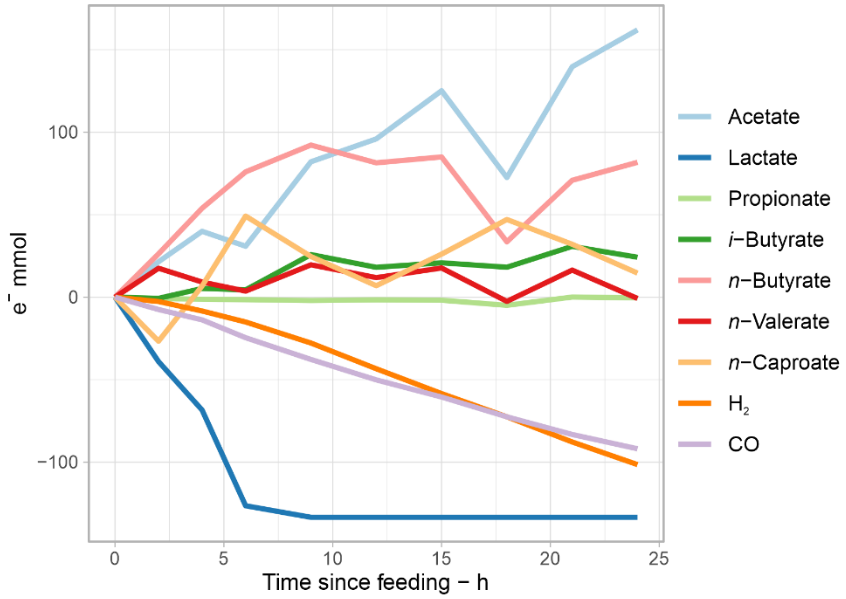

Fig. S3. Cumulative electron profiles of chemicals over the feeding cycle of day 42 in Reactor 1.

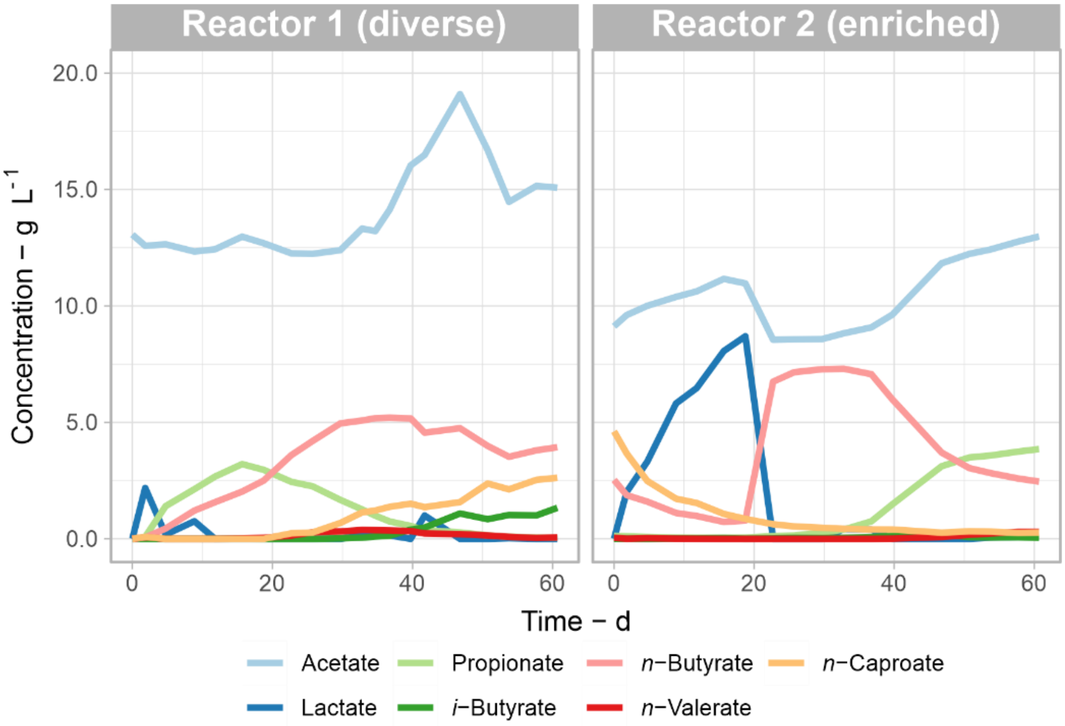

Fig. S4. Concentration profiles of carboxylates during the first 61 days of fermentation in Reactor 1 inoculated with a diverse community and Reactor 2 inoculated with an enriched culture.

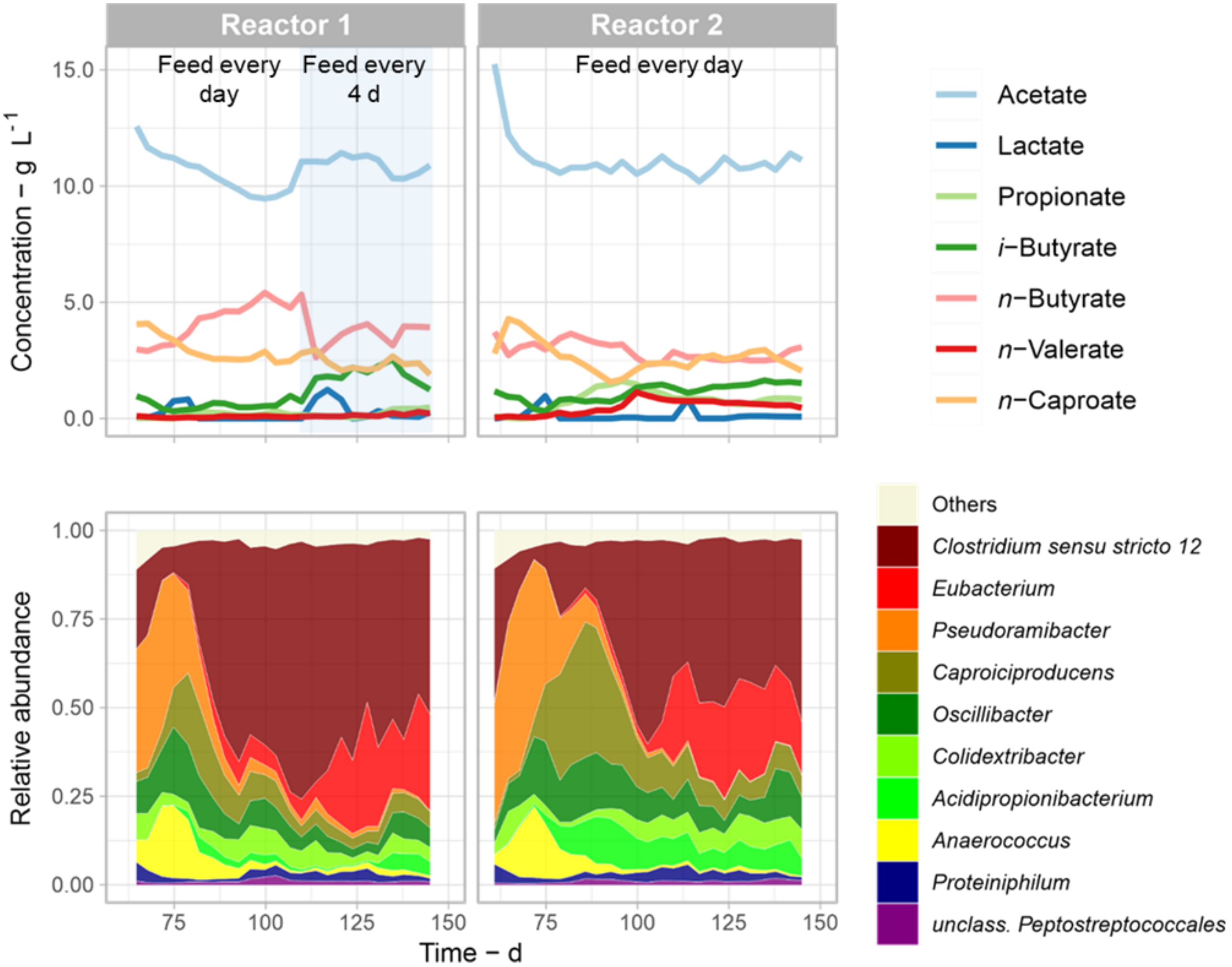

Fig. S5. Concentration of chemicals and community composition at the genus level between days 61 and 148. Between days 61 and 110, both reactors were fed once a day. Afterwards, Reactor 1 was fed once every four days, whereas the feeding regime of Reactor 2 remained unchanged. No clear effect of longer feeding intervals was seen.

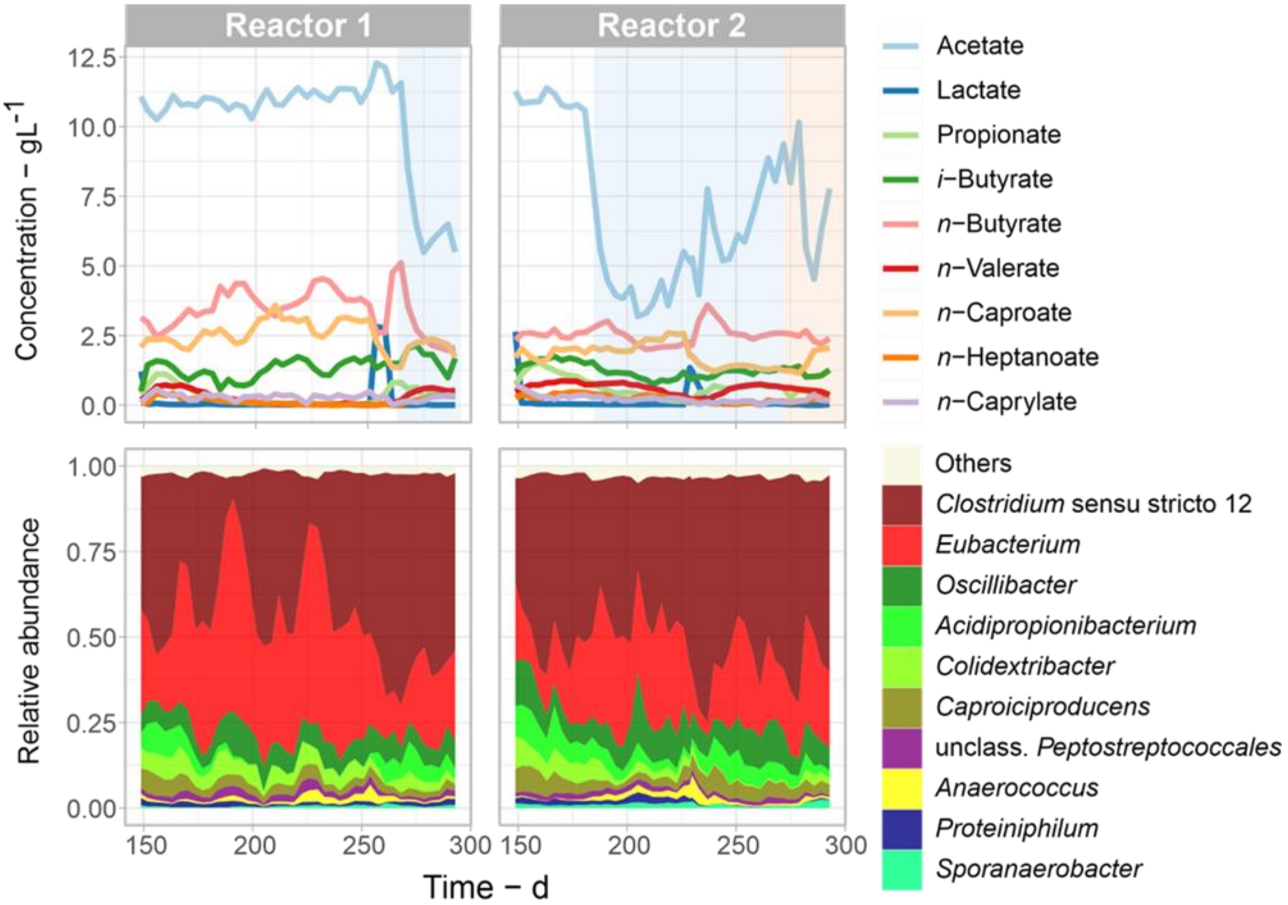

Fig. S6. Concentration of chemicals and community composition between days 148 and 292. The blue shading represents the period when acetate-free medium was fed. The period with a decrease of HRT to 10 d is shown with orange shading.

EFFECTS OF CHANGING OPERATING CONDITIONS. During the period of 61 – 148 d, both reactors showed similar community dynamics, even when the feeding interval was changed from 1 to 4 days in Reactor 1 on day 110 (Fig. S5). Excess acetate from the previous operation period (Reactor 1, Fig. S4) was washed out until it reached a relatively stable concentration of about 11 g L^{-1}. The highest concentration of *n*-caproate in the study (4.1 and 4.3 g L^{-1} *n*-caproate in Reactors 1 and 2, respectively) was observed in the period succeeding the acetate peak (Figs. S4 and S5). The *n*-caproate peak coincided with a steep increase in the relative abundances of *Pseudoramibacter* and *Anaerococcus* in both reactors. However, these two genera were only transiently detected in the community, as *Caproiciproducens*, *Clostridium* sensu stricto 12, *Oscillibacter*, and *Eubacterium* became more abundant from day 75. Between days 75 and 110, the *n*-caproate concentrations settled at around 2.5 g L^{-1} and the most distinctive

feature between the two reactors was a higher propionate concentration coinciding with higher abundance of *Acidipropionibacterium*, the putative propionate producer.

In comparison to the first 61 days of fermentation with the diverse community (Reactor 1, Fig. 1), the period between days 110 and 148 in Reactor 1 (feeding interval 4 d) showed net acetate consumption (13.6 e^- mmol L^{-1} d^{-1}), a decrease in gas consumption by 40% (to 45.2 e^- mmol L^{-1} d^{-1} or 554 mL L^{-1} d^{-1} of H_2 + CO), and an increase in elongation to *n*-caproate by about 47% (to 45 e^- mmol L^{-1} d^{-1}) (Reactor 1, Fig. S7A). For the following experiments, both reactors were operated with a feeding interval of 3.5 days (feeding twice per week) for ease of operation.

Reactor 2 received an acetate-free medium after day 181 (Fig. S6) causing higher gas consumption rates (84.0 e^- mmol H_2+CO L^{-1} d^{-1}) than Reactor 1 for the same period (34.2 e^- mmol H_2+CO L^{-1} d^{-1}) as seen in Fig. 2A (Acetate-free feed and 200 mM acetate, respectively). Ceasing acetate supply in Reactor 2 caused 42 e^- mmol L^{-1} d^{-1} to be routed from substrates to acetate in contrast to Reactor 1 that kept consuming 10 e^- mmol acetate L^{-1} d^{-1}. Consequently, the productivity of C≥4 carboxylates (i.e. *n*-butyrate, *i*-butyrate, *n*-valerate, *n*-caproate, *n*-heptanoate, and *n*-caprylate) in Reactor 2 decreased by 25% (from 132 to 98.9 e^- mmol L^{-1} d^{-1}) after acetate feeding had been stopped (Fig. 2A).

Interrupting acetate supply greatly increased carbon fixation rates in both reactors but decreasing HRT did not show a clear effect on fixation rates. Once both reactors were operating without acetate supply, a carbon fixation rate of 31.0 C mmol L^{-1} d^{-1} was seen in Reactor 1 with an HRT of 14 d and 28.0 C mmol L^{-1} d^{-1} was seen in Reactor 2 with an HRT of 10 d during the same period (Fig. 2B). Net carbon fixation was majorly due to CO consumption since CO_2 was a net product under all conditions. Net consumption of CO_2 can likely be achieved, if a syngas mixture with less CO is to be used.

The decrease of HRT from 14 to 10 d (i.e. a 40% dilution rate increase) corresponded to a 41% increase in the production rates of C≥4 carboxylates (from 111 to 156 e^- mmol L^{-1} d^{-1}, Fig. 2A) pointing out that the system could be optimized for higher rates without loss of selectivity to longer-chain carboxylates.

REFERENCES

Baleeiro, F.C.F., Ardila, M.S., Kleinsteuber, S. and Sträuber, H. (2021) Effect of Oxygen Contamination on Propionate and Caproate Formation in Anaerobic Fermentation. Front Bioeng Biotechnol 9, 725443.

Baleeiro, F.C.F., Kleinsteuber, S. and Sträuber, H. (2022) Recirculation of H_2, CO_2, and Ethylene Improves Carbon Fixation and Carboxylate Yields in Anaerobic Fermentation. ACS Sustainable Chemistry & Engineering.

Paradis, E., Claude, J. and Strimmer, K. (2004) APE: Analyses of Phylogenetics and Evolution in R language. Bioinformatics 20(2), 289-290.

Quast, C., Pruesse, E., Yilmaz, P., Gerken, J., Schweer, T., Yarza, P., Peplies, J. and Glockner, F.O. (2013) The SILVA ribosomal RNA gene database project: improved data processing and web-based tools. Nucleic Acids Res 41(Database issue), D590-596.

Schliep, K., Potts, A.J., Morrison, D.A., Grimm, G.W. and Fitzjohn, R. (2017) Intertwining phylogenetic trees and networks. Methods in Ecology and Evolution 8(10), 1212-1220.

Wright, E.S. (2015) DECIPHER: harnessing local sequence context to improve protein multiple sequence alignment. BMC Bioinformatics 16, 322.
